# A Rare Case of Myofibroma of the Mandible Complicated by Traumatic Neuroma Arising After a Conservative Surgical Approach

**DOI:** 10.4317/jced.62278

**Published:** 2025-05-01

**Authors:** Gabriel Pereira Rosa, José Renato Brandão, Thiago Pires Claudio, Matheus Antoni da Silva Costa, Rogério de Oliveira Gondak, Elena Riet Correa Rivero, Ricardo Luiz Cavalcanti de Albuquerque-Júnior

**Affiliations:** 1 Graduate Student, School of Dentistry, Federal University of Santa Catarina, Florianópolis/SC, Brazil; 2 MSc., School of Dentistry, Tiradentes University, Aracaju, Sergipe, Brazil; 3 MSc. Student, Post-Graduating Program in Dentistry, Federal University of Santa Catarina, Florianópolis/SC, Brazil; 4 PhD. Department of Pathology, Federal University of Santa Catarina, Florianópolis/SC, Brazil

## Abstract

Myofibroma (MF) is an uncommon benign mesenchymal neoplasm composed of myofibroblasts. Traumatic neuroma (TN) is a non-neoplastic proliferative disorder of the nerve sheath in response to injury or surgery. Both are uncommon in the jaws. We present a case of a 39-year-old Afro-descendant woman with a unilocular radiolucent lesion in the posterior mandible, showing mild expansion and cortical disruption. Biopsy revealed proliferation of fascicularly arranged spindle cells strongly positive for α-SMA and podoplanin, with low Ki-67 (<5%), and the final diagnosis was central MF. Ten months after enucleation and curettage, persistent pain led to a second biopsy, which revealed proliferation of multiple hyperplastic nerve fascicles consistent with TN. The postoperative course was uneventful with 14-month follow-up. A discussion on the clinicopathological criteria for differential diagnosis of MF and other spindle cell tumors as well as the rare occurrence of post-surgical TN is also provided.

** Key words:**Mouth neoplasms, differential diagnosis, mandible, oral pathology.

## Introduction

Myofibromas (MF) are uncommon benign mesenchymal tumors originating from neoplastic spindle cells with a myofibroblastic phenotype. First identified as “juvenile fibromatosis” by Stout in the 1950s, these tumors have since been extensively studied, confirming their myofibroblastic nature (1). Although approximately one-third of MF cases occur in the head and neck, oral cavity involvement is rare, especially in intraosseous locations, where it typically presents as a painless, slow-growing, well-defined osteolytic lesion (2). Diagnosis is based on histopathological identification of neoplastic spindle cells arranged in fascicles around vascular structures, often exhibiting a hemangiopericytoma-like pattern, with immunohistochemical positivity for smooth muscle markers, including α-smooth muscle actin (α-SMA) (3).

Traumatic neuroma (TN) is a benign lesion of nervous tissue caused by trauma or damage to a peripheral nerve, resulting in disorganized axonal growth during nerve regeneration. It commonly arises from direct nerve injuries, such as lacerations, compressions, or surgical trauma, and can occur at any site with peripheral nerves (4). TN clinically presents as a painful, touch-sensitive lesion, often accompanied by paresthesia or neuropathic pain in the area innervated by the affected nerve, microscopically characterized by disorganized proliferation of nerve fibers, including axons and Schwann cells along with fibrous tissue. Diagnosis is based on correlation of clinicopathological findings (5). Although TN is one of the most common neurogenic tumors of the oral and maxillofacial region, studies on the incidence of intraosseous traumatic neuromas after surgical excision of neoplastic or tumor-like conditions of the jaws are limited (6).

Herein, we report a rare case of myofibroma of the mandible managed by a conservative surgical approach with the subsequent development of a traumatic neuroma. A discussion of the clinicopathological and immunohistochemical findings of both lesions, along with the diagnosis-related challenges is also provided.

## Case Report

**Figure 1:**
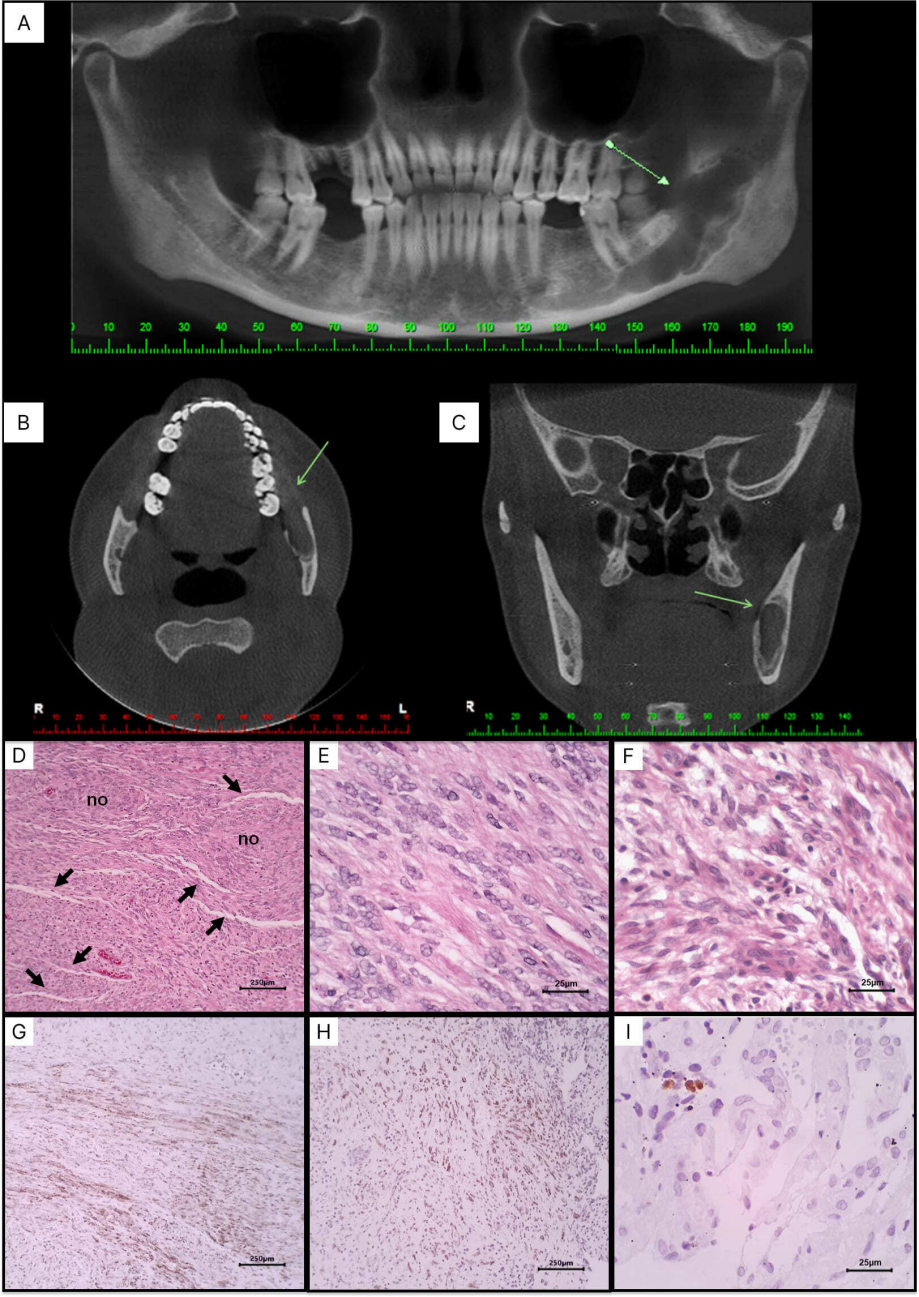
(A) Panoramic reconstruction, (B) Axial and (C) coronal sections of cone beam computed tomography showing an unilocular hypodense lesion in the posterior region of the mandible causing thinning and discontinuity of the cortical bone (arrows). Photomicrographs of histological sections showing (D) spindle-shaped to ovoid cells arranged in fascicular arrangement, and forming poorly delimited nodules (no), permeated by narrowed, thin-walled (“slit”) blood vessels (HE, 100 x). (E) Tumor cells exhibit either indistinct cytoplasm and vesicular nuclei (HE, 400 x) or (F) broad cytoplasm with imprecise boundaries, and elongated nuclei with dispersed chromatin (HE, 400 x). (G) Tumor cells are diffusely positive for α-SMA (SABCm 100 x) and (H) podoplanin (SABC, 100 x), (I) but only focally for Ki-67 (SABC, 400x).

A 39-year-old Afro-descendant woman was referred to a private dental clinic for implant placement. Extraoral examination revealed normal findings, including symmetrical facial thirds without lymphadenopathy. Intraoral examination showed good dental and periodontal health, despite the absence of teeth 16, 36, and 46. The patient was non-alcoholic, a non-smoker, and her medical and family history were non-contributory. Cone beam computed tomography revealed a well-defined osteolytic lesion with a sclerotic halo, extending from the apical region of tooth 37 to the coronoid process, causing thinning and discontinuity in the buccal and lingual cortical plates (Fig. 1A-C). Aspiration of the lesion was negative for fluid content. Suspecting intraosseous neoplasia, an incisional biopsy was performed. Histological analysis revealed a biphasic proliferation of rounded to spindle-shaped cells. Some cells exhibited indistinct cytoplasm, while others had broader cytoplasm with imprecise boundaries. Nuclei were elongated with dispersed and sometimes vesicular chromatin. Tumor cells formed interlaced fascicles of varying lengths. Narrow, thin-walled, slit-shaped blood vessels, surrounded by tumor cells, were frequently observed. The stroma showed fibrous tissue with focal hyalinization, myxoid changes, rare mitotic Figures, and no atypia or necrosis (Fig. 1D-F). Immunohistochemical analysis revealed cytoplasmic positivity for α-actin smooth muscle (α-SMA) and podoplanin in approximately 75% of the tumor cells, whereas Ki67 showed nuclear reactivity in less than 5% (Fig. 1G-I). Based on the correlation of clinicopathologic and immunohistochemical findings, the final diagnosis was central MF. The lesion was surgically enucleated, followed by bone curettage, and the patient remained under follow-up for eight months, showing satisfactory clinical evolution.

**Figure 2:**
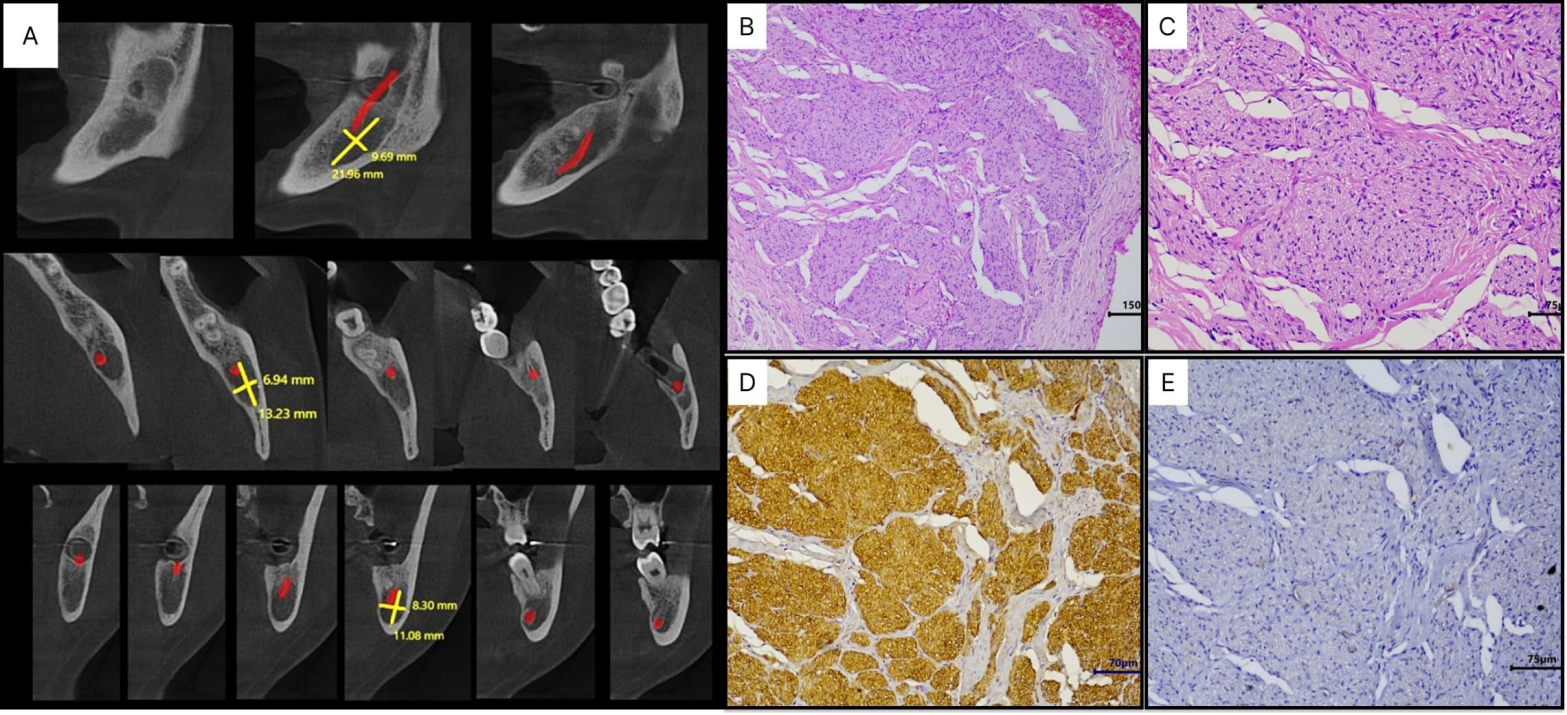
(A) Cone beam CT-scan showing a residual hypodense area measuring 8.3 x 13.2 mm at its largest diameter 10 months post-surgery. (B) Histological slides showing proliferation of peripheral nerve fascicles irregularly distributed in a fibrous connective tissue (SABC, 100 x), (C) composed of axons, surrounded by Schwann cells and perineural cells (SABC, 200 x). (D) Proliferative cells showing immunohistochemical positivity for S100 protein and (E) negativity for α-SMA (SABC, 200 x).

**Figure 3:**
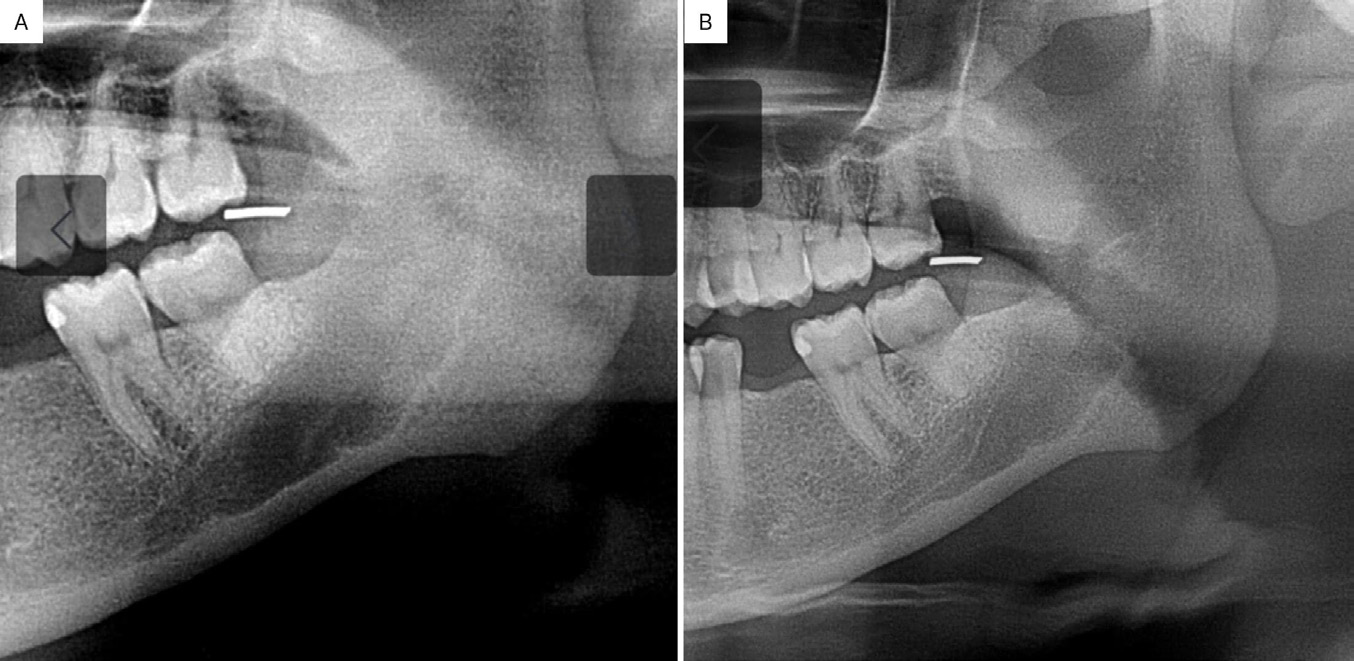
(A) Panoramic radiograph demonstrating evidence of progressive new bone formation at 12- and 14-months post-surgery.

Over the next two months, she reported severe pain in the mandibular region. Cone beam CT scan revealed a residual hypodense lesion, measuring 8.3 x 13.2 mm at its largest dimensions (Fig. 2A). With suspicion of TN, an excisional biopsy was performed. Histopathological analysis revealed disordered peripheral nerve fascicles composed of axons surrounded by Schwann and perineural cells within fibrous connective tissue. Immunohistochemical analysis revealed intense positivity for S-100 protein and negativity for α-SMA, confirming the diagnosis of TN (Fig. 2B-E). The patient remains under follow-up, with no signs of recurrence or pain, 14 months post-surgery (Fig. 3).

## Discussion

MFs are rare benign neoplasms of spindle cells with myofibroblast-like features, recently reclassified by the WHO as perivascular neoplasms likely originating from pericytes, forming a morphological continuum with myopericytoma, which (7). These tumors are categorized into three clinical forms: solitary, multicentric with visceral involvement, and multicentric without visceral involvement (8). Solitary intraosseous MF in the maxillofacial region, as reported herein, is rare. A systematic review reported by Silveira *et al*. (2024) showed that these tumors are more common in adult females, often presenting as asymptomatic swelling predominantly affecting the mandible (2), these findings align with the current case. Moreover, as occurred in the current case, incidental detection of the tumor during routine examinations, in the absence of apparent clinical signs or symptoms, has been previously reported (9). Furthermore, although MF typically presents as an osteolytic lesion with well-defined borders; however, larger lesions may cause cortical plate expansion, thinning, and perforation, suggesting potentially aggressive behavior (10), as observed in the current case.

Histopathologically, MF is a well-circumscribed neoplasm exhibiting a biphasic growth pattern. The central region contains spindle-shaped tumor cells associated with branching blood vessels resembling hemangiopericytoma, while the peripheral area consists of variably hyalinized, myoid-like cells (3). Although these features are consistent with the current case, they overlap with those of other spindle cell tumors, such as neurofibromas and solitary fibrous tumors (SFT). However, the immunohistochemical positivity for α-SMA confirmed the myofibroblast phenotype. Furthermore, the low proliferation rate (assessed by Ki-67), the low mitotic index and the absence of necrosis or cell atypia supported the exclusion of a malignant tumor (Souza *et al*., 2024). Tumor enucleation followed by curettage is the preferred method for managing well-encapsulated myofibromas (9). In contrast, non-encapsulated or infiltrative tumors require excision with clear margins to minimize the risk of recurrence (10). However, no recurrence was reported in any of the 19 cases, likewise the current case, attesting to the low rates of recurrence.

TN is a reactive lesion resulting from trauma or surgical intervention, often involving sensory nerve damage, due to disorganized axonal proliferation in the proximal segment of a severed nerve. Intraosseous development of TN, however, is rare, with few cases reported in the jaws; patients may present with localized pain, paresthesia, or altered sensation, though, some cases are asymptomatic (6), and it appears as a non-expansile, well-defined unilocular or ill-defined multilocular radiolucent lesion on imaging (11). In the current case, clinical and radiological findings suggested TN, likely due to trauma to the inferior alveolar nerve during myofibroma extraction. Histologically, the lesion exhibited non-encapsulated, disorganized nodules and nerve fascicles within a collagenous and fibroblastic stroma, typical of TN. Strong immunohistochemical positivity for S-100 protein confirmed the neural origin of the proliferative spindle cells, and the correlation between clinical, pathological, and immunohistochemical findings substantiated the diagnosis of TN. The therapeutic approach for this case was surgical excision of the neuroma. However, up to 42% of patients may experience persistent symptoms, requiring further interventions such as excision with neurorrhaphy, with or without nerve grafting (12). Recently, stem cell and tissue bioengineering therapies have emerged as potential options for recurrent TN (5). Additionally, rare cases of spontaneous remission have been reported (13). Preventive measures during surgery include minimizing nerve traction, promptly transecting the nerve stump, or cauterizing it with an electric scalpel (14).

In conclusion, although rare, MF should be included in the differential diagnosis of benign and low-grade malignant jaw lesions. Accurate diagnosis requires correlation between clinicopathological and immunohistochemical findings. Conservative surgical excision is the preferred treatment, but precise technique is essential to avoid nerve damage and complications, such as TN, which may require further intervention. This case emphasizes the importance of postoperative care and future research on surgical techniques and regenerative therapies could improve outcomes and reduce complication rates in similar cases.

## Data Availability

The datasets used and/or analyzed during the current study are available from the corresponding author.
